# Human serum inhibits adhesion and biofilm formation in *Candida albicans*

**DOI:** 10.1186/1471-2180-14-80

**Published:** 2014-03-28

**Authors:** Xiurong Ding, Zhizhong Liu, Jianrong Su, Donghui Yan

**Affiliations:** 1 Clinical Laboratory Center, Beijing Friendship Hospital, Capital Medical University, Beijing 100050, China; 2 The Centre for Laboratory Diagnosis, Beijing Tiantan Hospital, Capital Medical University, Beijing 100050, China

**Keywords:** Human serum, Biofilm, Adhesion, *Candida albicans*

## Abstract

**Background:**

*Candida albicans* can form biofilms on intravenous catheters; this process plays a key role in the pathogenesis of catheter infections. This study evaluated the effect of human serum (HS) on *C. albicans* biofilm formation and the expression of adhesion-related genes *in vitro*. A *C. albicans* laboratory strain (ATCC90028) and three clinical strains were grown for 24 h in RPMI 1640 supplemented with HS or RPMI 1640 alone (as a control). The growth of biofilm cells of four strains was monitored by a Live Cell Movie Analyzer, and by XTT reduction assay. The expression of the adhesion-related genes *BCR1*, *ALS1*, *ALS3, HWP1* and *ECE1* was analyzed by RT-PCR at three time points (60 min, 90 min, and 24 h).

**Results:**

In the adhesion phase, *C. albicans* cells kept a Brownian movement in RPMI medium containing HS until a large number of germ tubes were formed. In the control group, *C. albicans* cells quickly adhered to the bottom of the reaction plate. Compared with RPMI 1640, medium supplemented with 3–50% HS caused a significant decrease in biofilm development (all *p* < 0.001). However, the presence of HS had no significant inhibitory effect on the pre-adhered biofilms (all *p* > 0.05). Biofilm formation was also inhibited by heat-inactivated and proteinase K pre-treated HS. The presence of 50% HS did not significantly affect the planktonic growth of C. albicans (*p* > 0.05). At three time points, HS inhibited expression of the *ALS1* and *ALS3* genes and promoted expression of the *HWP1* and *ECE1* genes. Significant up-regulation of *BCR1* was observed only at the 90-min point.

**Conclusions:**

Human serum reduces biofilm formation by inhibiting the adhesion of *C. albicans* cells. This response may be associated with the down-regulation of adhesion-related genes *ALS1*, *ALS3* and *BCR1*. The inhibitory serum component is protease-resistant and heat stable.

## Background

*Candida* spp. are the fourth most common cause of nosocomial bloodstream infections [1], and *Candida albicans* accounts for approximately 50% of cases of candidemia [2]. Frequently, candidemia is associated with *C. albicans* colonization of indwelling devices, such as catheters, endotracheal tubes, and pacemakers [3-6]. In fact, *C. albicans* is the most common fungus in biofilms formed on medical devices [7]. Biofilm formation is a complex, multicellular process, consisting of cell adhesion, growth, morphogenic switching between yeast and filamentous states, and quorum sensing [8,9]. Adhesion of *C. albicans* cells to materials or host cells is a prerequisite for biofilm formation, and cell-cell interactions may be important in the hierarchical organization of cells within the biofilm [6]. Moreover, biofilm formation of *C. albicans* is governed by a tightly woven gene network composed of six transcription regulators and their target genes [10]. The zinc finger transcription factor *BCR1* and its target genes, *ALS1*, *ALS3*, *HWP1*, and *ECE1*, play an important role, especially in the process of adhesion [11-13].

Human serum (HS) is a complex medium composed of proteins, lipids, and small molecules. The interaction of *C. albicans* with serum has been of long-standing interest in the field of fungal pathogenesis. Because *Candida* spp. can form biofilms on intravenous catheters and other inserted medical devices that may come into contact with blood, serum is regarded as an external cue to trigger biofilm formation. Yuthika *et al.*[14] reported that 3% human serum can promote the formation of *C. albicans* biofilms. However, other researches revealed that serum can inhibit biofilm formation in some bacteria. Another study showed that human serum and fetal bovine serum (FBS) inhibit biofilm formation in *Staphylococcus aureus*[15], and Hammond *et al.*[16] found that adult bovine serum (ABS) or adult human serum (AHS) also inhibits *P. aeruginosa* biofilm formation on plastic surfaces, including intravenous catheters. Some studies revealed the ability of serum components to prevent the formation of bacterial biofilms. It was reported that bovine serum albumin (BSA) caused a significant decrease in biofilm development [16]. Abraham *et al.* indicated that a low molecular weight component of human serum inhibits biofilm formation in *Staphylococcus aureus*[15]. In addition, one component of innate immunity also prevents bacterial biofilm development [17].

Therefore, our hypothesis is that the positive effect of human serum on *Candida albicans* biofilm formation may be due to many factors, so it is necessary to study the related molecular mechanism.

## Results

### The *C. albicans* adhesion process

To directly observe the adhesion process of *C. albicans*, the Live Cell Movie Analyzer was used. For the first 2 or 3 h of biofilm formation, we took photos once per minute by means of continuous photographic techniques. When those pictures were played back in rapid succession, we got dynamic images of biofilm growth. Movie 1 shows that cells of *C. albicans* quickly adhered to the surface of polypropylene microtiter plates, formed germ tubes, and gradually extended in RPMI 1640 without HS (Additional file 1: Movie 1). However, in the RPMI 1640 with 50% HS, the cells of the same strain kept a Brownian motion at the beginning and could not quickly clung to the bottom of the plate. The Brownian motion lasted as long as about 2 h. The motion did not stop until the formation of a large number of germ tubes (Additional file 1: Movie 2). In the next hour (120–180 min), almost no *C. albicans* cells kept a Brownian motion, but the hyphae grew longer (Additional file 1: Movie 3). Movie 3 further shows that Brownian motion stops after 2 h (Additional file 1: Movie 1, Movie 2, and Movie 3).

### Effect of human serum on germ tube formation of *C. albicans*

*C. albicans* cells were cultured in RPMI 1640 with and without 50% HS, and germ tube formation was continuously observed at 30, 60, 90, 120, and 180-min time points by Live Cell Movie Analyzer. For the first 90 min of culture, the germ tube formation rate of *C. albicans* cells in the experimental group (RPMI 1640 containing 50% human serum) was significantly lower than that in the control group. Over 2 h of incubation, there was no significant difference in the rate of germ tube formation between the two groups. With the further extension of incubation time (from 2 h to 3 h), the amount of hyphae gradually increased in the experimental group, just as in the control group (Additional file 2).

### Effect of human serum on *C. albicans* biofilms

Data comparing biofilm growth of *C. albicans* strains in the absence or presence of different concentrations of HS were obtained using a XTT reduction assay. Initially, the tests were performed using cells of strain ATCC90028 in RPMI 1640 containing different concentrations of HS (3%, 5%, 10%, and 50%).

**Figure 1:**
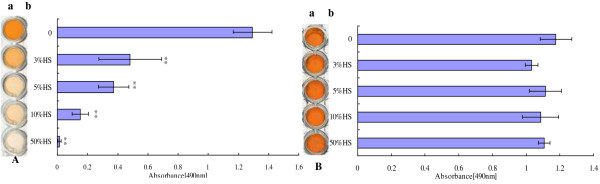
**Effect of human serum on *C. albicans *biofilms. A)** Analysis of biofilm formation in the presence of normal human serum (HS). ATCC90028 was grown in polypropylene microtiter plates at 37°C for 24 h in the presence of different concentrations of HS. **a**. Scanned image of the XTT reduction assay for quantitation of biofilms. **b**. Quantitation of biofilms by XTT reduction assay. **B)** Different concentrations of HS were added to pre-adhered biofilms of ATCC90028 and incubated in RPMI 1640 medium for an additional 24 h at 37°C. **a**. Scanned image of the XTT reduction assay for quantitation of biofilms. **b**. Quantitation of biofilms by XTT reduction assay. All experiments were done in triplicate with three technical repeats on separate days with similar results and shown as a representative image. RPMI 1640/HS *vs*. RPMI 1640, **p < 0.01.

It was found that HS inhibited the biofilm formation of *C. albicans* in a dose-dependent manner (from 3% to 50%). More specifically, 3% HS was sufficient to inhibit biofilm formation (*p* < 0.001), and this anti-biofilm effect increased with increasing HS concentrations (Figure 1A). However, HS had no significant inhibitory effect on pre-adhered *C. albicans* biofilms *in vitro* (all *p* > 0.05), even when the concentrations were as high as 50% (Figure 1B).

To confirm the hypothesis that this effect was not specific to strain ATCC90028, we tested three unrelated clinical strains and found that HS also had the same effect on all three clinical strains (data not shown).

### Characterization of the inhibitory components

**Figure 2:**
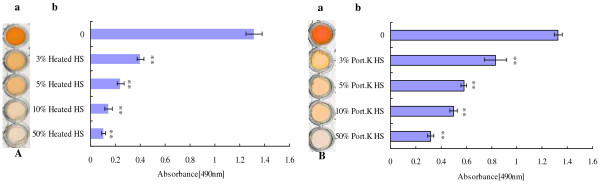
**The component(s) of serum inhibit *C. albicans *biofilm formation. A)** Biofilm formation of *C. albicans* ATCC90028 was examined in the presence of different concentrations of heat-inactivated human serum for 24 h at 37°C. **a**. Scanned image of the XTT reduction assay for quantitation of biofilms. **b**. Quantitation of biofilms by XTT reduction assay. **B)** Biofilm formation of *C. albicans* ATCC90028 was examined in the presence of different concentrations of proteinase K-treated human serum for 24 h at 37°C. (**a**. Scanned image of the XTT reduction assay for quantitation of biofilms. **b**. Quantitation of biofilms by XTT reduction assay.) All experiments were done in triplicate with three technical repeats on separate days with similar results. RPMI 1640/HS *vs.* RPMI 1640, **p < 0.01.

To further investigate the component(s) of serum that affect the adhesion of *C. albicans*, we heated the serum at 56°C for 30 min. This heat treatment did not abrogate the inhibitory activity. Heat-inactivated serum still inhibited biofilms in a dose-dependent manner (Figure 2A). At a concentration of 3%, heat-inactivated HS significantly inhibited biofilm formation (*p* < 0.001), and with increasing HS concentrations, the effect of HS on biofilm formation became more pronounced. To eliminate the possibility that a heat stable protein was responsible for the biofilm inhibition, proteinase K was used to degrade proteins in the HS, but this also did not affect the ability of serum to inhibit biofilm formation (Figure 2B). Biofilm formation was significantly reduced in proteinase K-treated serum compared with the control group (all *p* < 0.001). At a concentration of 3%, proteinase K-treated HS significantly inhibited biofilm formation (*p* < 0.001), and with increasing HS concentrations, the effect of HS on biofilm formation became more pronounced. The results were similar in all four *C. albicans* strains (data not shown).

### Effect of human serum on planktonic growth of *C. albicans*

**Figure 3:**
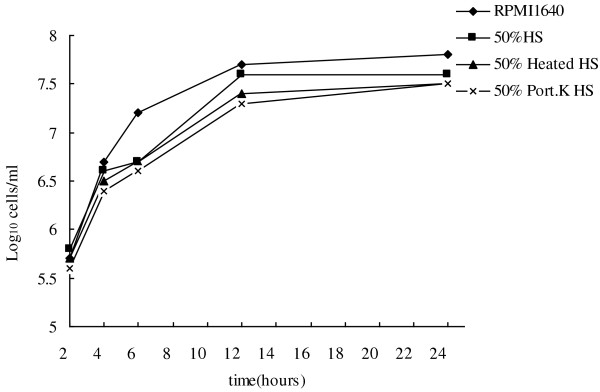
**Effect of human serum on planktonic growth of *C. albicans. *** Twenty-four-hour growth curves showing 50% HS, 50% heat-inactivated HS, and 50% proteinase K-treated HS against *C. albicans* ATCC90028 in RPMI 1640. Symbols: ◆, growth control; ■, 50% HS; ▲, 50% heated HS; ×, 50% proteinase K-treated HS.

To confirm that inhibition of biofilm formation was not due solely to growth inhibition, the effect of HS on the planktonic growth of *C. albicans* was investigated. Time-growth curves indicated that the presence of 50% HS (fresh HS, heat-inactivated HS, or proteinase K-treated HS) did not significantly affect the growth of *C. albicans* (all *p* > 0.05) (Figure 3). To confirm the hypothesis that this effect was not specific to strain ATCC90028, we tested three unrelated clinical strains and found that HS had the same effect on all three clinical strains as well (data not shown).

### Effect of human serum on expression of adhesion-related genes

**Figure 4:**
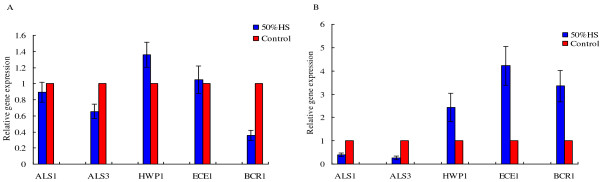
**Expression of *C. albicans *adhesion-related genes. ***Candida albicans* cells were incubated in the absence or presence of HS (50%) and the expression of target genes was determined by RT-PCR. Housekeeping gene *ACT1* was used as an internal control. Each gene was assessed in triplicate, and the experiment itself was performed in biologic duplicate. The data shown here are a representative graph of strain ATCC90028. **A)** Expression of genes *ALS1*, *ALS3*, *HWP1*, *ECE1*, and *BCR1* following the treatment with HS for 60 min. **B)** Different expression of the target genes following treatment with HS for 90 min. **C)** Target gene expression level following treatment with HS for 24 h.

To elucidate the potential molecular mechanism behind the ability of HS to prevent growth of *C. albicans* biofilms, total RNA was isolated from biofilms of four *C. albicans* strains grown in RPMI 1640 medium with or without 50% HS at three time points (60 min, 90 min and 24 h). The expression levels of specific genes that were previously implicated in mediating the adhesion of *C. albicans* cells were determined by real-time RT-PCR. HS had varying effects on different genes in different tested strains (data not shown), but the general trend of these genes was consistent. HS down-regulated the expression of the adhesion-related genes *ALS1* (1.1 to 3.0-fold) and *ALS3* (1.5 to 3.8-fold), but up-regulated the expression of the hypha-related genes *HWP1* (1.1 to 2.4-fold) and *ECE1* (1.1 to 4.2-fold) at all three time points (Figure 4). Particularly, expression levels of *ALS1* (2.5 and 3.0-fold) and *ALS3* (3.7 and 3.8-fold) showed significant differences at both 90 min and 24 h (*p* < 0.05 or *p* < 0.01) (Figure 4B,C). Only at the 90-min time point were the transcription levels of *HWP1* (2.4-fold) and *ECE1* (4.2-fold) significantly higher (*p* < 0.05 or *p* < 0.01) (Figure 4B). The transcription level of *BCR1* was significantly higher at 90 min (3.3-fold, *p* < 0.01) (Figure 4B), but *BCR1* levels were significantly lower at both 60 min (2.8-fold, *p* < 0.05) and 24 h (5.6-fold, *p* < 0.01) (Figure 4A,C).

## Discussion

To make the transition from a commensal organism to a systemic pathogen, *C. albicans* must first enter the bloodstream. It can do so by taking advantage of medical devices, such as intravenous catheters, to enter the bloodstream directly, or it can cross intact or damaged gastrointestinal mucosa and enter into the bloodstream [5,18]. In the bloodstream, *C. albicans* is exposed to the innate immune defenses. As a part of human innate immune system, serum and its components show different degrees of protection against systemic candidiasis.

In this study, the natural proliferation condition of *C. albicans* was monitored continuously by a Live Cell Movie Analyzer. *C. albicans* cells in HS moved with Brownian motion in the initial stage of culture, then failed adhere to the surface of polystyrene plates. This indicates that *C. albicans* may remain in a suspended status at the early period of entering the blood stream. Previous studies showed that free-flowing *C. albicans* can be rapidly cleared from the blood [19]. We determined that human serum facilitates the removal of *C. albicans* by inhibiting the adhesion of *C. albicans* on the surface of the endothelial cells. *C. albicans* possesses virulence factors that are needed to establish candidiasis that are involved in the many steps of this complicated process, such as adhesion, phenotypic switching, morphogenesis, and biofilm formation [20]. Some factors in the bloodstream, such as temperature and serum, facilitate the filamentation of *C. albicans*[21-23]. It is reported that filamentation is favorable for *C. albicans* adhesion and biofilm formation [24,25]. However, filamentation failed to offset the biofilm formation inhibition caused by the HS-induced adhesion defect, as demonstrated in our study.

We also investigated the effect of serum on germ tube formation in *C. albicans*. Our results showed that the rate of germ tube formation is high in HS medium, but compared with the control group, germ tube formation in the experimental group was delayed in the initial stage of culture (within 90 min). This may be one of the reasons for HS-induced adhesion inhibition. Based on these results, we also think that RPMI 1640 medium may be a more suitable medium than human serum to conduct germ tube testing in *C. albicans*.

In the initial stage of biofilm culture (the adhesion period), low serum concentrations suppressed *C. albicans* biofilm formation. However, the serum had no effect on pre-adhered biofilms (90 min), even if the serum was in a very high concentration. Thus, we concluded that serum may inhibit biofilm formation by preventing the adhesion of *C. albicans*, in consensus with previous studies [15,16]. Recent studies showed that addition of as little as 3% human serum to media can promote *C. albicans* biofilm formation [14], contrary to our results. This may be explained by the use of different materials, such as serum, culture medium, strains, adhesive medium, and so on.

It has been reported that IgG, LL-37, transferrin and lactoferrin, at concentrations close to those found *in vivo*, can reduce the capacity of *C. albicans* and bacteria to adhere to polystyrene [17,26-28]. Thus, we initially hypothesized that some protein or antimicrobial peptide in human serum may inhibit the adhesion of *C. albicans*, and as a consequence, reduce biofilm formation. However, our results suggest that the compound in serum that inhibits *C. albicans* biofilm formation is not proteinaceous. Abraham *et al.*[15] found that a low molecular weight component of human serum inhibits biofilm formation in *Staphylococcus aureus,* and the component was protease-resistant and heat stable. We conclude here that human serum may also contain non-protein component(s) that can inhibit the adhesion and biofilm formation of fungi and bacteria. To confirm this hypothesis, future studies are needed to identify this component of human serum.

In this study, planktonic growth of *C. albicans* was not inhibited by human serum, indicating that inhibition of biofilm formation was not due solely to growth inhibition.

Biofilm formation of *C. albicans*, a process that depends upon both cell-cell and cell-substrate adherence, is controlled by a tightly woven network of genes [10]. Among this gene network, *BCR1* is one of the best-characterized biofilm regulators [11-13,29]. Through its adhesin targets *ALS1, ALS3*, *HWP1* and *ECE1, BCR1* mediates cell-substrate and cell-cell interactions in biofilms [30,31]. In this study, at the adhesion stage of biofilm formation (60 min, 90 min), the expression of *BCR1* went from less than to significantly higher than that of the control group. This may be due to the promoting effect of serum on hypha growth, as *BCR1* RNA accumulation depends on the hyphal developmental activator *TEC1*[32]. *ALS1* and *ALS3* are members of the agglutinin-like sequence (*ALS*) gene family that encodes cell-wall glycoproteins [33]. Most Als proteins have adhesin functions [34,35]. Mutational analysis indicates that strains lacking all functional *ALS1* and *ALS3* alleles (*als1Δ/als1Δ als3Δ/als3Δ*) failed to produce any detectable adherent cells in biofilm models both *in vivo* and *in vitro*[30], or in actual biofilm formation. The *als1Δ/als1Δ* mutants produced substantial biofilms, but the biofilms often sloughed off the substrate, while the *als3Δ/als3Δ* mutant only produced scant, disorganized biofilms on catheter material *in vitro*[12]. Our data on transcript analysis showed that the expression of *ALS1* and *ALS3* were reduced at different time points in the biofilm adhesion stage. Therefore, we supposed that the anti-adhesion effect of human serum might occur via inhibition of the expression of *ALS1* and *ALS3*, and therefore affect biofilm formation. Previous studies have shown that a *bcr1Δ*/ *bcr1Δ* mutant, which has reduced expression of *ALS1*, *ALS3*, and other adhesins, has defective biofilm formation in both an *in vitro* and *in vivo* catheter model [12]. In this study, at 90 min of growth, the change in the levels of *BCR1* level was different from *ALS1* and *ALS3*, indicating that *ALS1* and *ALS3* are also affected by other factors [8,36].

Interestingly, human serum promotes the expression of *HWP1* and *ECE1. HWP1* is a well-characterized hypha-specific gene that can mediate *C. albicans* cell-cell interactions and improve biofilm formation [37,38]. Nobile *et al.*[30] found that the expression of Hwp1 in *Saccharomyces cerevisiae* permits adherence to wild-type *C. albicans* but not an *als1Δ/als1Δ als3Δ/als3Δ* double mutant. In addition, a *TDH3-HWP1* hybrid gene could not promote biofilm formation in the *als1Δ/als1Δ als3Δ/als3Δ* background *in vitro* or *in vivo*. Our study revealed that human serum decreased the expression level of *ALS1* and *ALS3,* so overexpression of *HWP1* failed to save the adhesion and biofilm formation of *C. albicans. ECE1* was regarded as a hyphal-induced gene, although its mechanism of action is uncertain. Our study showed that hyphae were significantly greater in the presence of serum than in the control group, especially in the mature biofilm stage (data not shown). This may be due to the increase of *ECE1* and *HWP1*[23].

In this study, we also tested the expression of adhesion-related genes in biofilms grown for 24 h and found that the expression trend of related genes at this time was similar to the adhesion phase, both in the reduction of *ALS1* and *ALS3* and the up-regulation of *HWP1* and *ECE1*. The expression of the *BCR1* gene, however, was significantly inhibited. Its level was far lower than that of the control group. All in all, the serum reduces *BCR1* gene expression, and that might be a reason for biofilm inhibition.

## Conclusion

In summary, our study demonstrated that human serum may reduce the biofilm formation of *C. albicans* by inhibiting adhesion. This inhibition is partly due to the down-regulation of adhesion-related genes, including *ALS1*, *ALS3* and *BCR1*. Meanwhile, the inhibitory effect of human serum is caused by non-protein components in the serum. Therefore, biofilm formation *in vivo* may be “selected for” (possibly by immune pressure and sheer forces) rather than “induced” by serum at the level of transcription.

## Methods

### Ethics Statement

This study was approved by the Medical Ethics Committee of Beijing Friendship Hospital, Capital Medical University, Beijing, China (approval #BJFH-EC/2013-014), and individual informed consent was waived.

### Organisms

Four *Candida albicans* strains (laboratory strain ATCC90028 and three clinical isolates of *C. albicans*: 9079, y2991, 31448) were tested in this study. The three *C. albicans* bloodstream isolates were collected from three different intensive care patients admitted to the Beijing Friendship Hospital and were confirmed according to standard mycological methods, such as the germ tube test in serum, growth on CHROMagar *Candida* medium, and API testing methods. All isolates were stored in skim milk at -80°C until use.

### Medium and growth conditions

Prior to each experiment, *C. albicans* strains were subcultured on Sabouraud's Agar (SDA) at 35°C for 24 h. To prepare the yeast inocula for biofilm growth, a loopful of the SDA culture was transferred into 25 ml of liquid yeast extract-peptone-dextrose (YPD) medium (1% yeast extract, 2% peptone, 2% glucose) and incubated at 30°C for 18 h in an orbital shaker (75 rpm). Then, the cells were harvested by centrifugation, washed twice in PBS (pH 7.2), re-suspended in RPMI 1640 medium (buffered to a pH of 7.0 with 0.165 M morpholinepropanesulfonic acid), and counted after serial dilution by a hemocytometer.

### Human serum

Human serum (HS) was pooled from healthy blood donors, and heat-inactivated serum was prepared by heating at 56°C for 30 min. Proteinase K-treated serum was prepared by incubating with 50 mg/mL proteinase K at 58°C for 1 h followed by incubation at 85°C for 1 h to inactivate the protease. All fractions were filter-sterilized (0.22-mm pore size filter).

### Biofilm formation

Fungal biofilms were prepared as described on commercially available, pre-sterilized, flat-bottomed 96-well polystyrene microtiter plates (Corning) [39]. Briefly, a cell suspension of 1.0 × 10^6^ cells/ml was prepared in RPMI 1640 and RPMI 1640 + 50%, 10%, 5% or 3% HS. From those suspensions, 100 μl was introduced into wells and incubated at 37°C for 24 h without agitation, which allowed the cells to attach to the surface of the plate and form the biofilm structure.

To investigate the effect of HS on pre-adhered biofilms, *C. albicans* biofilms were prepared for 90 min (the adhesion phase) at 37°C as described above. The wells were washed twice with PBS to remove loosely adherent cells. Then, fresh RPMI 1640 (100 μl), containing different concentrations (3–50%) of HS were added and the plate was further incubated for 24 h at 37°C. RPMI 1640 medium without HS was included in control wells. The metabolic activity of the *C. albicans* biofilms was determined quantitatively using XTT reduction assay.

### Dynamic monitoring of the adhesion process

Standard cell suspension of *C. albicans* was prepared in RPMI1640 or RPMI1640 containing different concentrations (3% to 50%) of HS, and 100 μl of those suspensions was introduced into 96-well polystyrene microtiter plates. After standing for 3 min, the plates were placed on Live Cell Movie Analyzer (JuLI^™^ Br., NanoEnTek Inc., Seoul, Korea) and incubated at 37°C. The instrument was set to continuous photographing mode with exposure 5%, brightness 13%, zoom level 4, interval 1 min, and total time 2 h (the experimental group was prolonged to 3 h). When it was finished, a total of 121 or 181 photos were obtained for the control and experimental groups, respectively. Then, those pictures were played back in rapid succession to observe the dynamic changes of the fungal cells (playing at a speed of 10 frames/s).

### Quantitation of biofilms

At the end of the incubation, the supernatant was aspirated and the wells washed twice with PBS. The quantitation of biofilms was determined using 2,3-bis (2-methoxy-4-nitro-5-sulfo-phenyl)-2H-tetrazolium-5-carboxanilide (XTT) reduction assay that measures the activity of mitochondrial dehydrogenase [40]. XTT solution (1 mg/ml) was prepared by dissolving XTT powder (Sigma, Shanghai, China) in PBS, and the solution was filter-sterilized (0.22-mm pore size filter). XTT solution (40 μl) was mixed with freshly prepared menadione solution (0.4 mM; 2 μl) (Sigma, Shanghai, China) at 20:1 (v/v) immediately prior to the assay. Thereafter, PBS (158 μl) was mixed with XTT-menadione solution (42 μl), transferred to each well containing pre-washed biofilms, and incubated in the dark for 3 h at 37°C. After the incubation, the colored supernatant (100 μl) was transferred to new microtiter plates, and the optical density of the supernatant was measured at 490 nm with a microplate reader (BIO-RAD, CA, USA) and imaged by a flatbed scanner (EPSON PERFECTION V700 PHOTO, Beijing, China). All assays were carried out in at least three replicates on different days.

### Effect of human serum on planktonic growth of *C. albicans*

A cell suspension of 10^5^ cells/ml was prepared in RPMI 1640, RPMI 1640 + 50% fresh HS, 50% heat-inactivated HS and 50% proteinase K-treated HS. At predetermined time points (0, 2, 4, 6, 12 and 24 h after incubation with agitation at 30°C), 100 μl aliquot was removed from every solution and serially diluted 10-fold in sterile water. A 100 μl aliquot from each dilution was streaked on the Sabouraud dextrose agar plate. Colony counts were determined after incubation at 30°C for 48 h. Three independent experiments were performed. Effect of human serum on growth of *C. albicans* was determined by analyzing the time-growth curve.

### RT-PCR analysis of *C. albicans* adhesion-related genes

Quantitative real-time reverse transcription PCR (RT-PCR) was used to compare mRNA abundances of the genes of interest. A standard cell suspension of *C. albicans* (1 ml) was transferred into the wells of a pre-sterilized, flat-bottomed 24-well polystyrene microtiter plate (Corning, NY, USA). After incubation for 60 min, 90 min or 24 h at 37°C with or without HS, the supernatant was aspirated and the wells were washed twice with PBS. Total RNA was extracted from *C. albicans* biofilms using FastPure™ RNA kit (TaKaRa Biotechnology Co. Ltd, Dalian, China), according to the manufacturer’s manual. RNA concentrations and RNA purity were determined using a BioPhotometer spectrophotometer (Eppendorf, Germany). An equal amount of RNA was subjected to cDNA synthesis using the PrimeScript RT reagent kit (TaKaRa Biotechnology Co. Ltd, Dalian, China).

Real-time PCR primers were designed for the target genes *ALS1*, *ALS3*, *ECE1*, *HWP1*, and *BCR1* using Primer Express 3.0 software (Applied Biosystems, CA, USA). The β-actin gene (*ACT1*) was used as an endogenous reference gene. The sequences of forward and reverse primers are shown in Table 1. Real-time RT-PCR was performed with a StepOnePlus™ real-time PCR system (Applied Biosystems, CA, USA), and SYBR® Premix Ex Taq™ II was used as a reagent specifically designed for intercalator-based real-time PCR using SYBR Green I. All PCR reaction mixtures contained: 10 μl SYBR® Premix Ex TaqTM II (2X), 2 μl first strand cDNA, 0.5 μl each primer, 0.4 μl ROX Reference Dye (50X) and dH_2_O to the final volume of 20 μl. The program for amplification was 95°C for 30 s as an initial denaturation step, followed by 40 cycles of PCR consisting of 95°C for 5 s and 60°C for 30 s. Negative controls (water as template) were included in each run. After amplification, a melting curve was analyzed to confirm the specificity of the primers. Expression of each investigated gene was normalized to the housekeeping *ACT1* gene and analyzed using comparative *C*t method (ΔΔ*C*t). Expression of *ALS1*, *ALS3*, *ECE1*, *HWP1*, and *BCR1* genes from cells grown under serum-treatment condition was indicated as relative expression to that of genes from untreated yeast cells. Each experimental condition was performed in duplicate and each experiment was repeated twice on two different days for reproducibility.

**Table 1:** Primers used for RT-PCR experiments

***Primer***	***Sequence***	***Tm (°C)***
*ALS1-F*	5’-CCTATCTGACTAAGACTGCACC-3’	57.69
*ALS1-R*	5’-ACAGTTGGATTTGGCAGTGGA-3’	60.13
*ALS3-F*	5’-ACCTGACTAAAACTGCACCAA-3’	57.71
*ALS3-R*	5’-GCAGTGGAACTTGCACAACG-3’	60.59
*HWP1-F*	5’-CTCCAGCCACTGAAACACCA-3’	60.18
*HWP1-R*	5’-GGTGGAATGGAAGCTTCTGGA-3’	60.00
*ECE1-F*	5’-CCCTCAACTTGCTCCTTCACC-3’	59.96
*ECE1-R*	5’-GATCACTTGTGGGATGTTGGTAA-3’	59.82
*Bcr1-F*	5’-GCATTGGTAGTGTGGGAAGTTTGAT-3’	57.64
*Bcr1-R*	5’-AGAGGCAGAATCACCCACTGTTGTA-3’	59.96
*ACT1-F*	5’-CGTTGTTCCAATTTACGCTGGT-3’	60.03
*ACT1-R*	5’-TGTTCGAAATCCAAAGCAACG-3’	58.01

### Statistical analysis

Data were described as mean ± SD. All statistical analyses were performed by statistical analysis computer software package SPSS 17.0 (SPSS Inc., IL, USA). Student’s *t*-test or one-way ANOVA were used to compare the biofilm formation, planktonic growth, and the gene expression of *C. albicans* strains in the presence or absence of HS. Results with a *p*-value less than 0.05 were considered statistically significant.

## Competing interests

The authors declare that they have no competing interests.

## Authors' contributions

XRD conceived and designed the experiments and carried out most of the data collection and drafted the manuscript. ZHZHL participated in data analysis and interpretation and drafted the manuscript. JRS conceived the study, participated in its design and revised the manuscript. DHY contributed to data analysis. All authors read and approved the final manuscript.

## Supplementary Material

Additional file 1 ***C. albicans *** **ATCC90028 was incubated in polypropylene microtiter plates at 37°C in the absence or presence of HS (50%) and the plates were placed on Live Cell Movie Analyzer.** The instrument was set to continuous photographing mode with exposure 5%, brightness 13%, zoom level 4, interval 1 min, and total time 2 h (the experimental group was prolonged to 3 h). Movie 1 Video of *C. albicans* biofilm grown in the RPMI 1640 without HS during the first 2 h (0–120 min). Movie 2 Video of *C. albicans* biofilm grown in the RPMI 1640 with HS during the first 2 h (0–120 min). Movie 3 Video of *C. albicans* biofilm grown in the RPMI 1640 with HS in 120–180 min. Click here for file

Additional file 2 **Light microscopy images of *C. albicans *** **ATCC90028 biofilms in RPMI and RPMI + HS media.** The different panels show photomicrographs taken at various time points during germ tube formulation, as indicated. Click here for file
