# Supplementary material for: Human serum inhibits adhesion and biofilm formation in Candida albicans
Source: BMC Microbiol. 2014 Mar 28;14:80. doi: 10.1186/1471-2180-14-80 (PMC4101872; doi:10.1186/1471-2180-14-80)
Supplement: Additional file 2 — Light microscopy images of C. albicans ATCC90028 biofilms in RPMI and RPMI + HS media. The different panels show photomicrographs taken at various time points during germ tube formulation, as indicated. [file 1471-2180-14-80-S2.doc]

RPMI 1640

RPMI 1640 containing 50% human serum


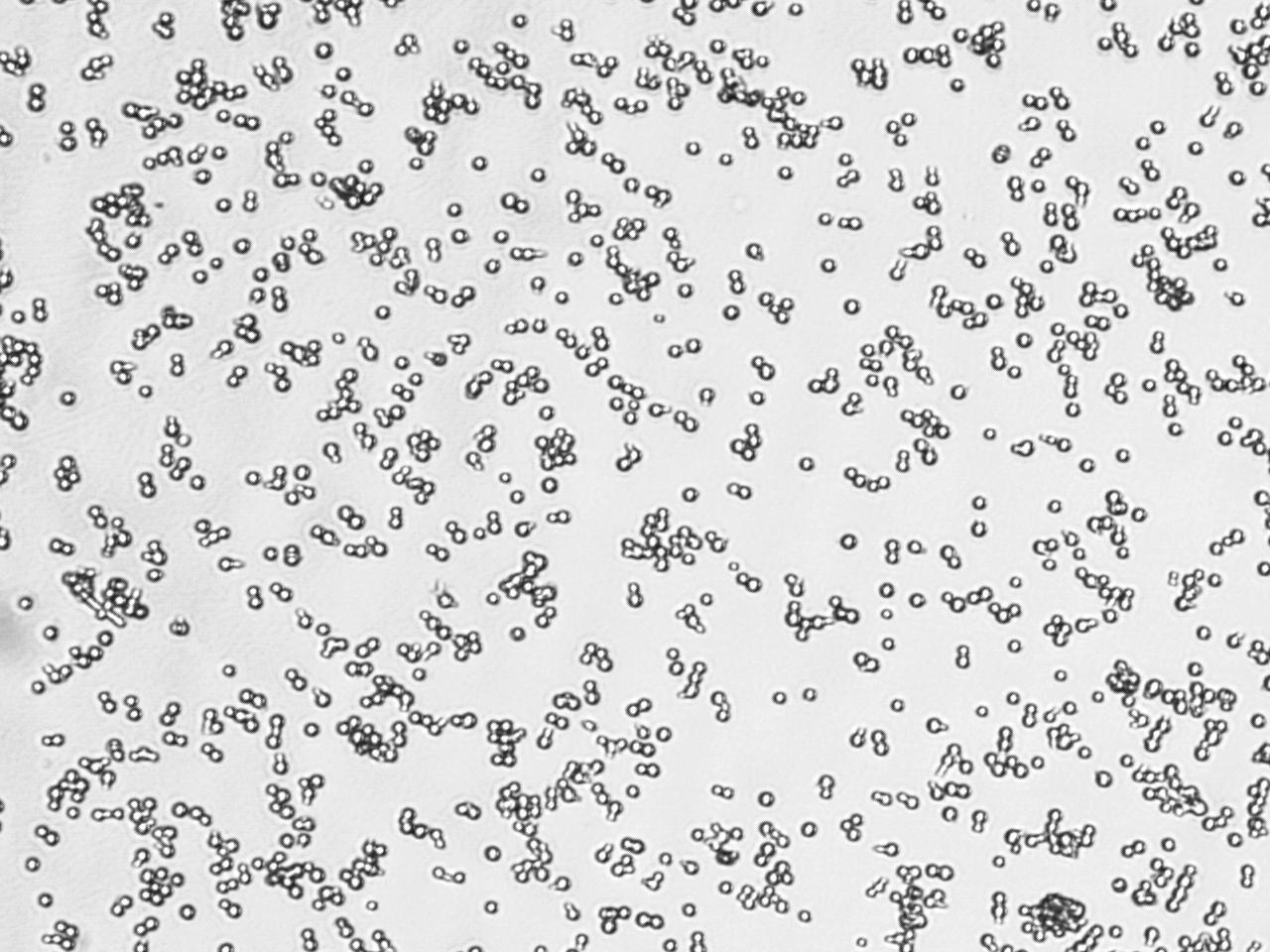

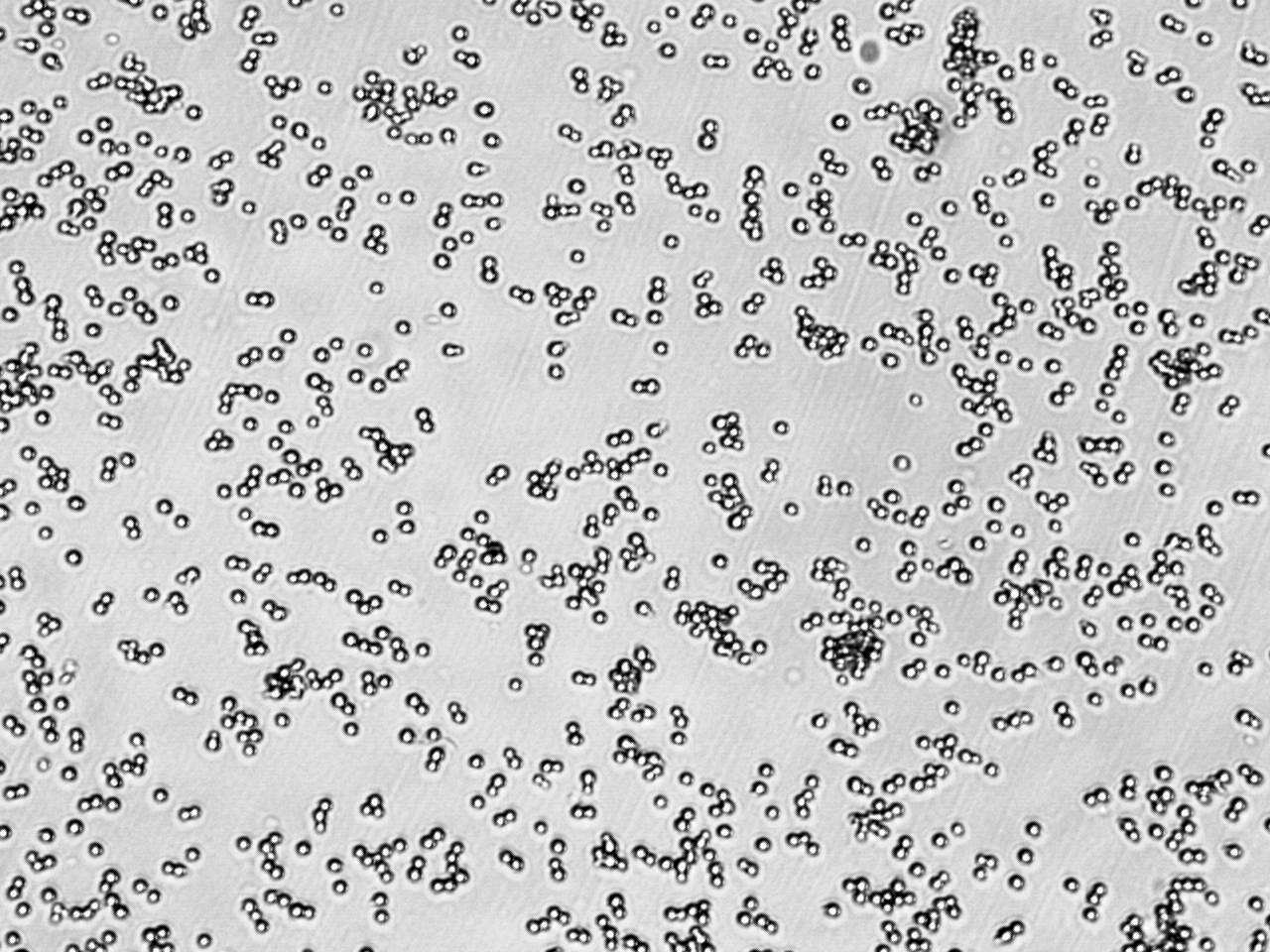
**30min**


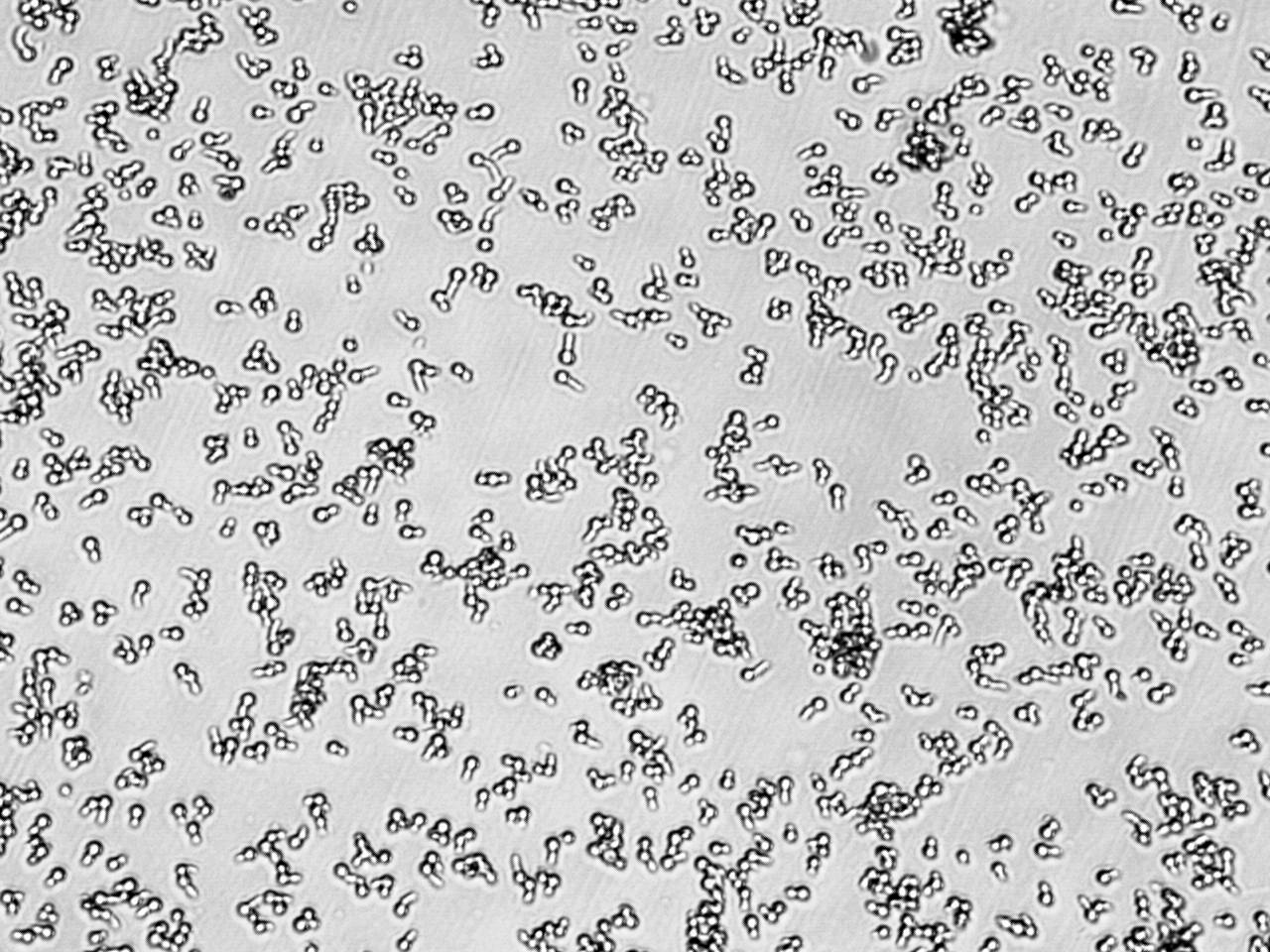
60min


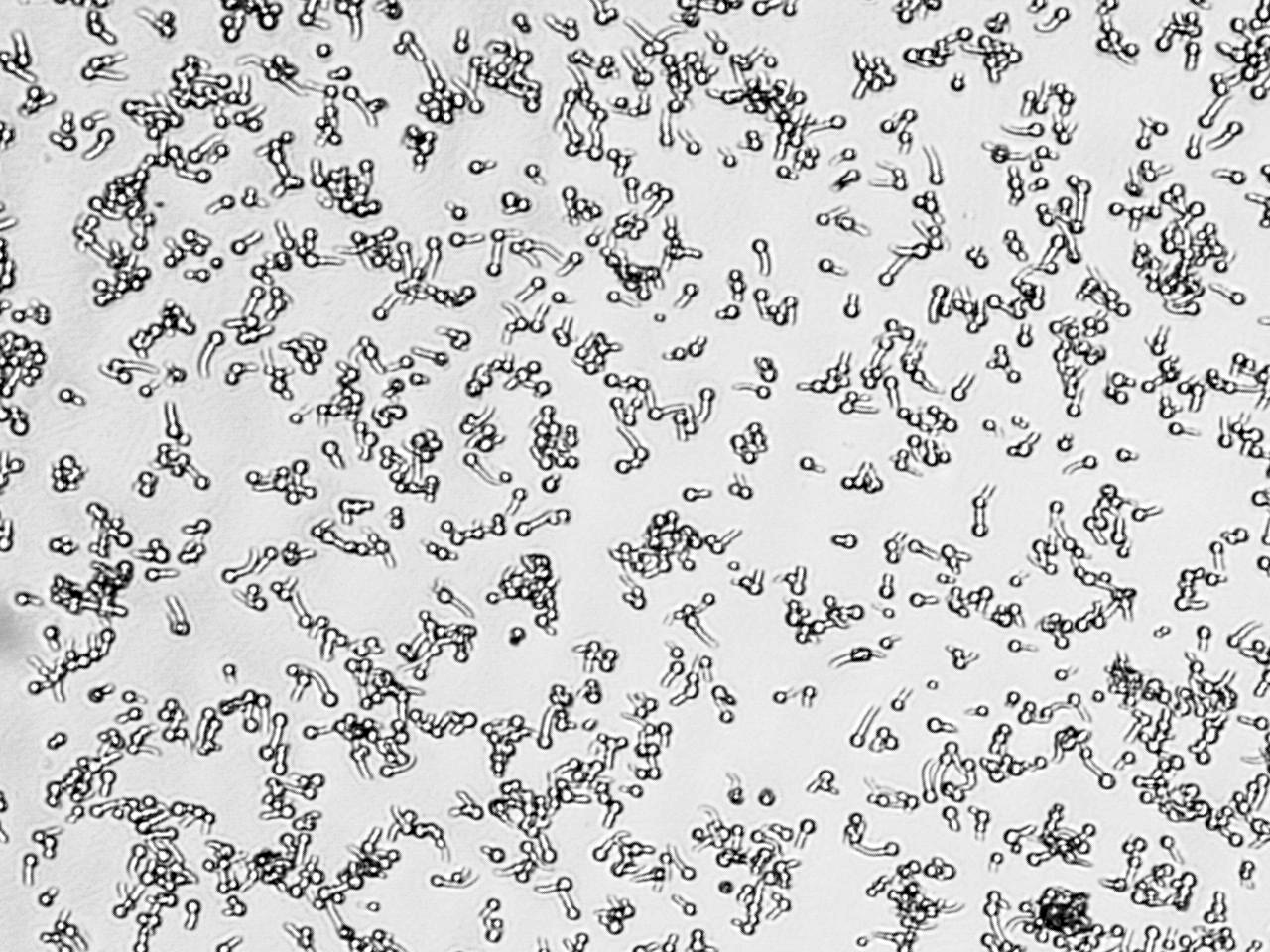


90min


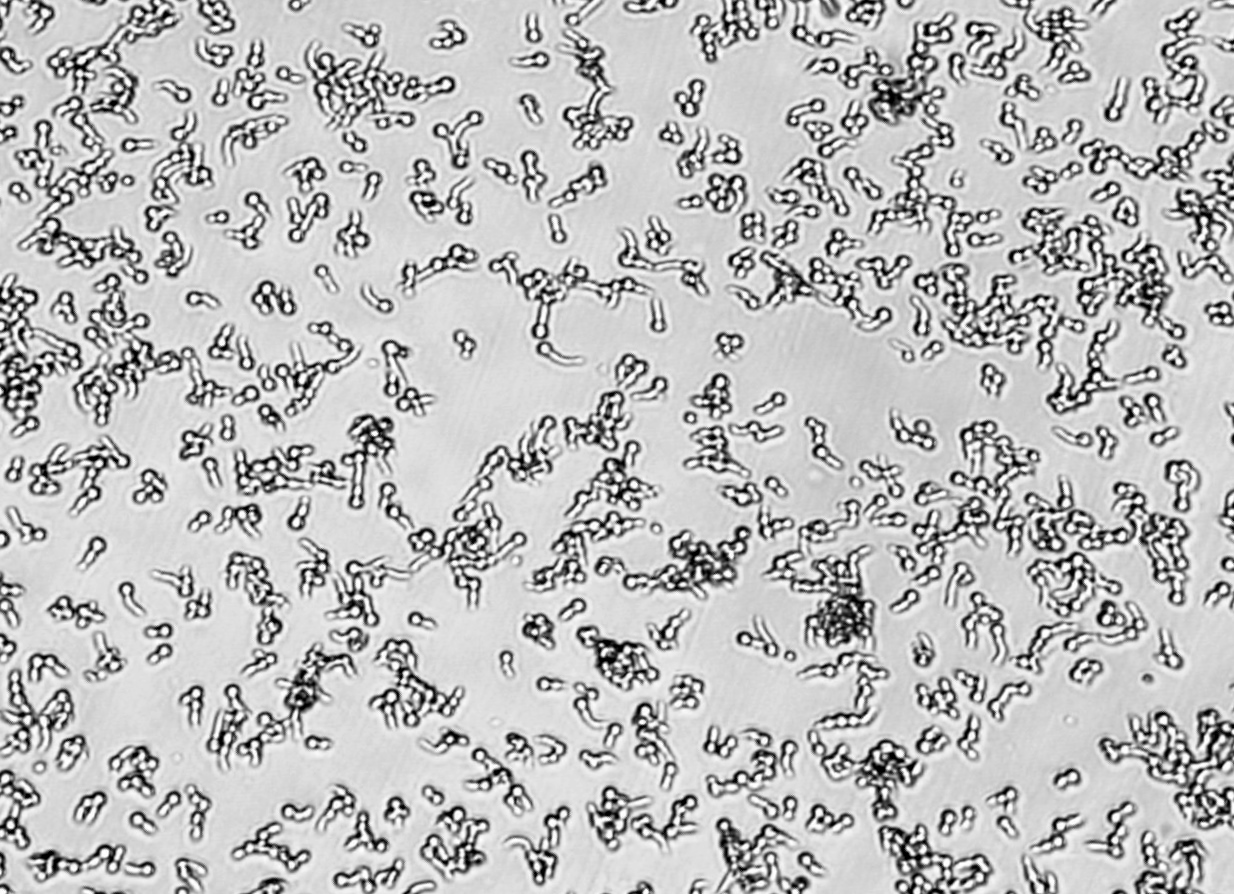

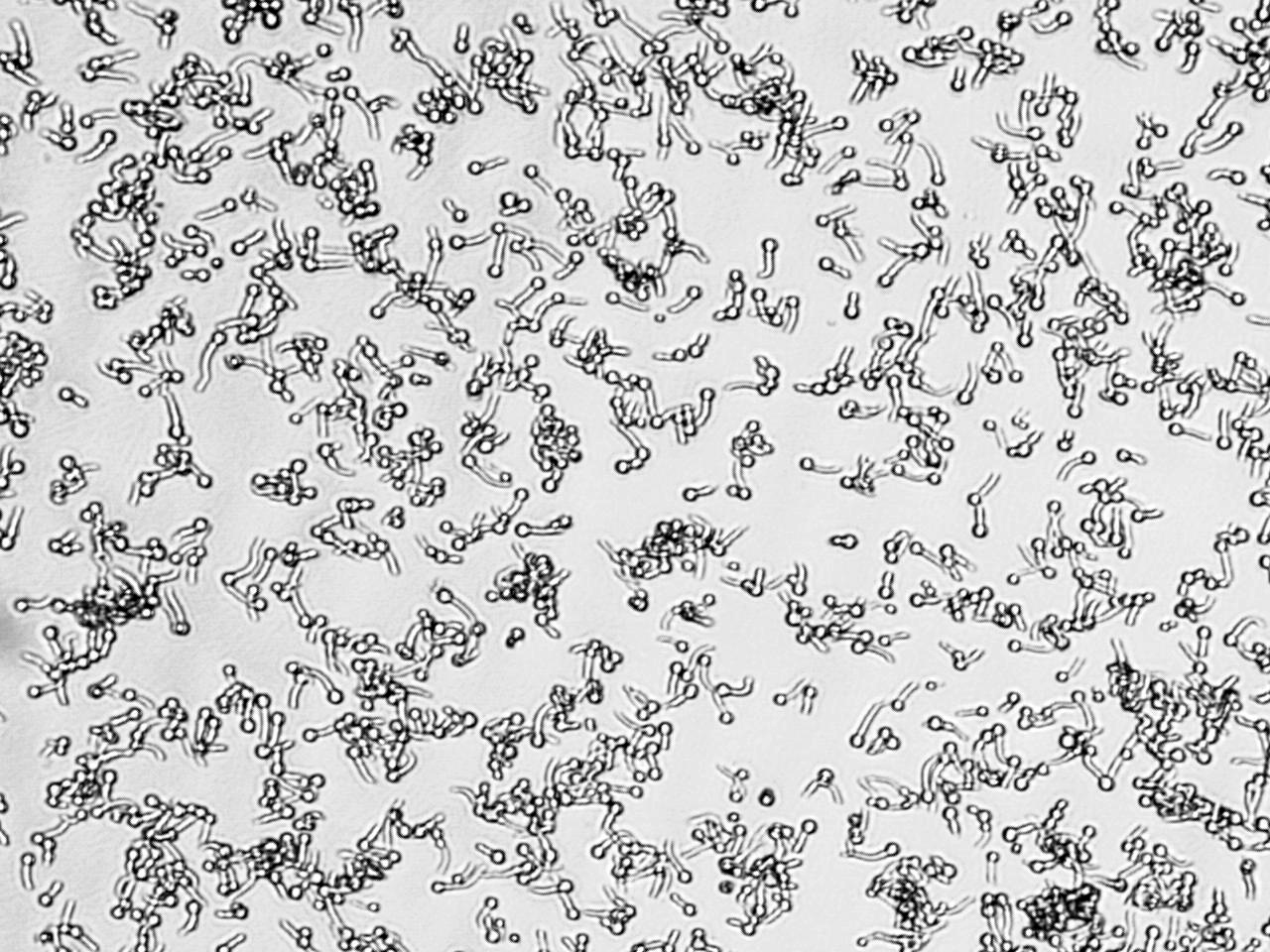


120min


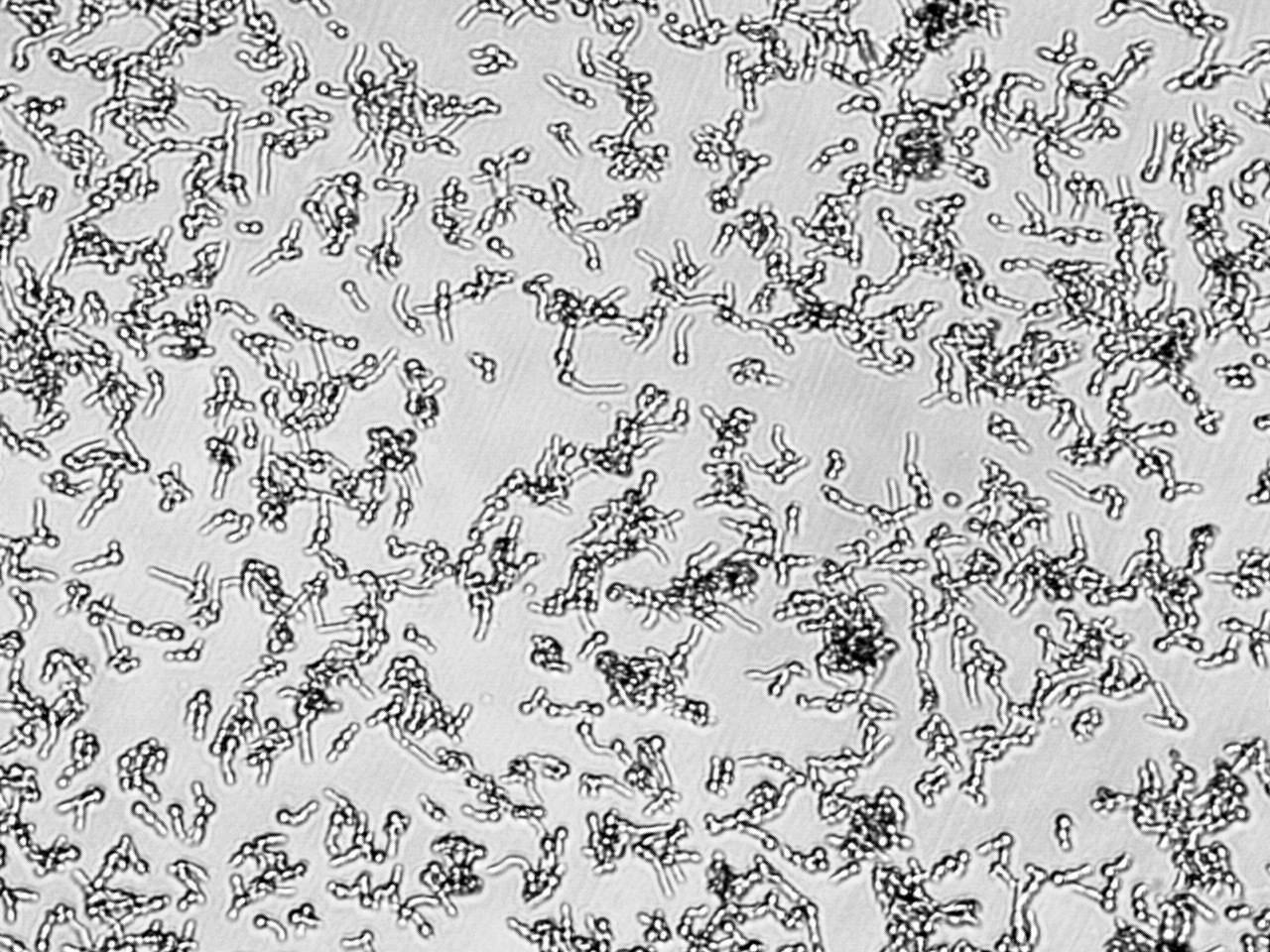

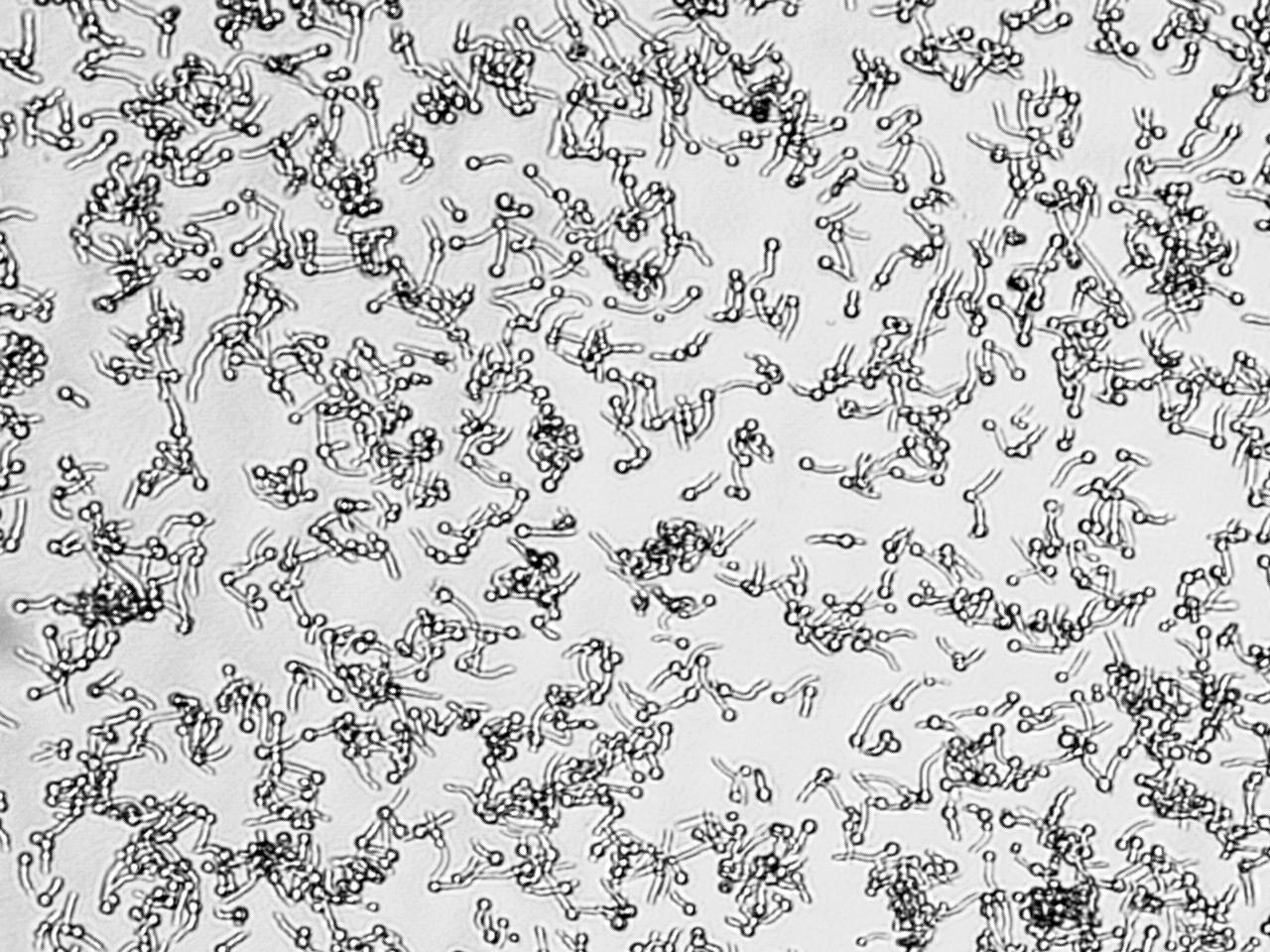


180min


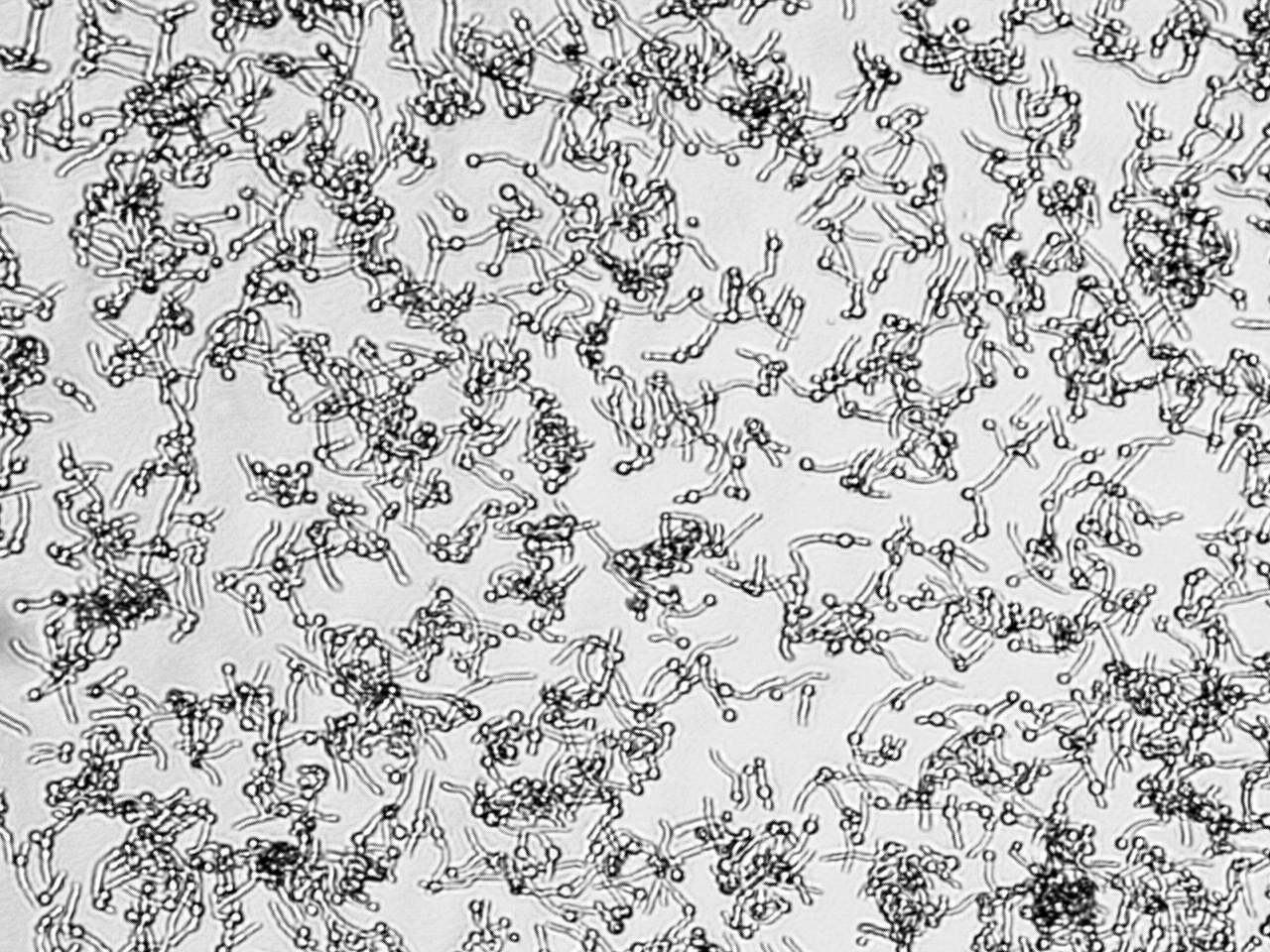

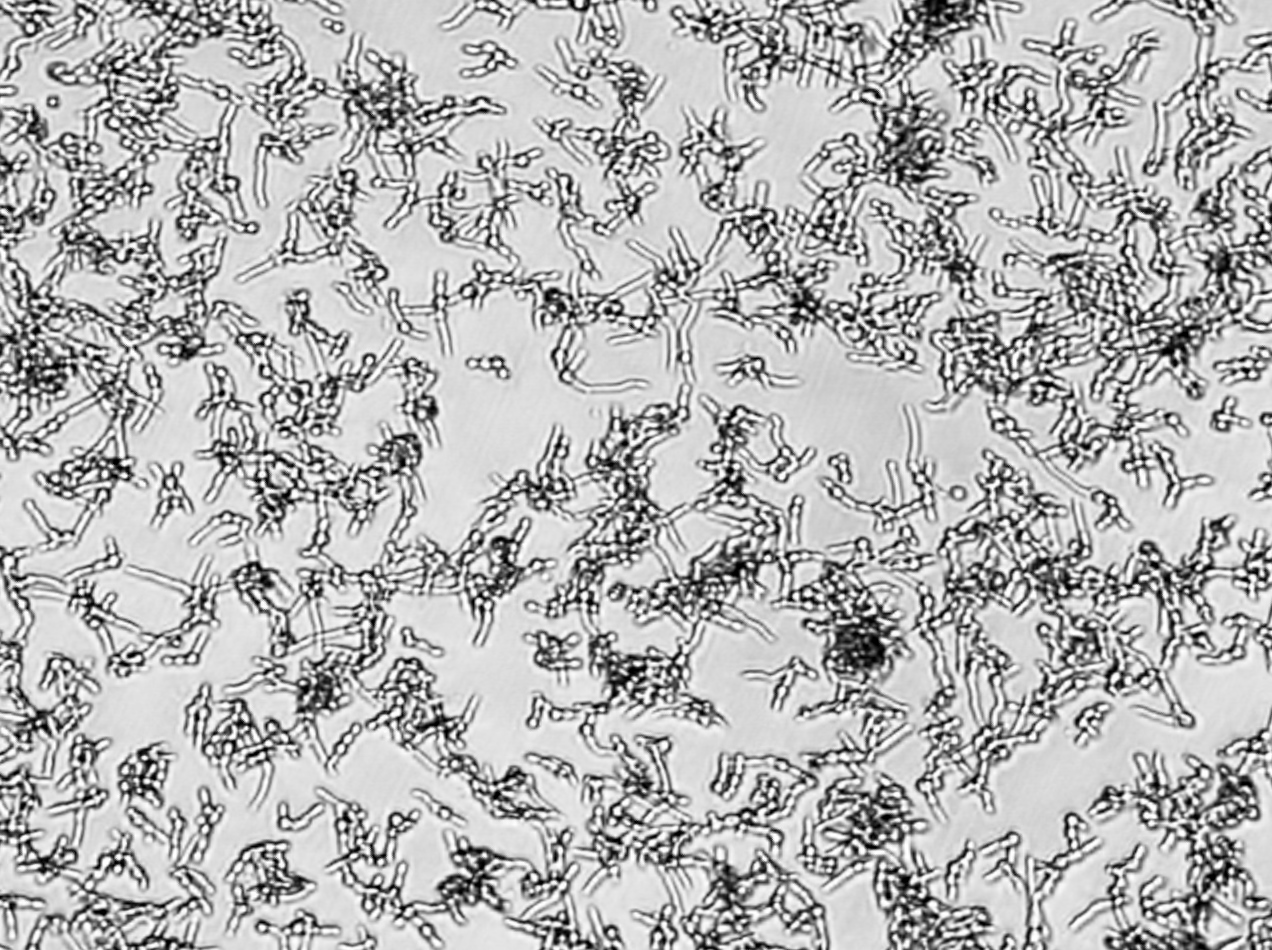


**supplementary material 2**. Light microscopy images of C. albicans ATCC22018 biofilms in RPMI and RPMI+HS media. The different panels show photomicrographs taken at various time points during germ tube formulation, as indicated.
